# Microbe Profile: Candidozyma auris: an emergent and resilient yeast and new antifungal strategies

**DOI:** 10.1099/mic.0.001664

**Published:** 2026-02-06

**Authors:** Gordon Ramage, Leighann Sherry, Ryan Kean

**Affiliations:** 1 School of Health and Life Sciences, Glasgow Caledonian University, Glasgow, UK; 2 School of Infection and Immunity, University of Glasgow, Glasgow, UK

**Keywords:** antifungal, biofilm, *Candidozyma auris*, resistance

## Abstract

*Candidozyma auris* is an emerging opportunistic yeast that is important because of its multidrug resistance, persistence in healthcare environments and ability to cause outbreaks. Since its discovery in 2009 in Japan, it has rapidly spread worldwide and is now recognized as a major global public health threat and was recognized by the World Health Organization (WHO) in 2022 as a critical priority fungal pathogen. Distinct phylogeographic clades demonstrate simultaneous emergence on different continents, suggesting ecological or environmental triggers. Clinical management is complicated by frequent resistance to fluconazole, reduced susceptibility to amphotericin B and echinocandins, and frequent misidentification by traditional laboratory methods. Continued genomic surveillance, improved diagnostics and new antifungal strategies are urgently needed, supported by enhanced infection prevention and control procedures.

## Graphical abstract

Overview of *Candidozyma auris* biology. (a) Climate change and associated environmental stressors are hypothesized to have been a driver for this heat and salt tolerant pathogen to emerge. Exactly how it spreads from the environment into the hospital remains unknown. (b) Within the clinical environment, it is able to colonize and persist on the skin of patients and clinical staff. It is readily transferred and shed from patients onto fomites and medical equipment, where it can survive for prolonged periods. (c) On patients and the wider hospital environment, it can persist as adherent wet and dry surface biofilm communities that are highly tolerant to antifungal agents, antiseptics and disinfectants through inhibition by biofilm properties (e.g. extracellular matrix).

## Taxonomy

Kingdom: Fungi; Phylum: Ascomycota; Subphylum**:** Saccharomycotina

Class: Pichiomycetes; Order: Serinales; Family: Metschnikowiaceae; Genus: *Candidozyma*; Species: *Candidozyma auris* (formerly *Candida auris*)

## Properties

*C. auris* grows as budding yeast cells and can tolerate elevated temperatures (up to 42 °C) and high salinity, aiding persistence in the environment and in hospitals. *C. auris* demonstrates strain- and clade-specific pathogenicity [1]. It also shows thermotolerance and halotolerance, enhancing both environmental and clinical persistence. Animal models confirm its ability to cause lethal systemic infections and adapt morphologically during host interactions. It forms antimicrobial-tolerant biofilms on abiotic surfaces and shows variable morphogenetic plasticity, producing pseudohyphae and filaments under stress conditions [2]. Colonization of skin and mucosal surfaces facilitates transmission, distinguishing it from most other *Candida* species. Little is known about how it survives in the environment.

## Genome and evolution

The haploid genome of *C. auris* is ∼12–13 Mb, encoding ~6000 genes across 5 to 7 chromosomes, and has a G+C content of 44.5–44.8% [3]. Comparative genomics highlights lineage-specific expansions of transporter families (Binding Cassette (ABC) transporters and major facilitator superfamily) and genes associated with stress resistance. Independent emergence of at least six clades across continents suggests convergent adaptation, possibly linked to environmental change.

## Phylogeny

Whole-genome sequencing has revealed six major clades: These include clade I (South Asian), clade II (East Asian), clade III (South African), clade IV (South American), clade V (Iranian) and the recently described clade VI (Indomalayan). Recent phylogenomic analyses prompted reclassification into the genus *Candidozyma*, reflecting its divergence from *Candida* species [4]. Phylogenetic studies confirm clonal spread within healthcare facilities but significant divergence between clades, supporting independent emergence. Between clades, there are single nucleotide polymorphism (SNP) differences in the tens of thousands. From within clades, differences between isolates are typically less than 100 SNPs.

## Key features and discoveries

*C. auris* was first described in 2009 in Japan [5] and remarkably emerged with several independent clades across different continents [6]. *C. auris* is frequently misidentified by conventional biochemical systems, often confused with *C. auris* appearing as *Candidozyma haemulonii*. Reliable identification requires updated matrix-assisted laser desorption/ionization–time of flight (MALDI-TOF) mass spectrometry databases or molecular sequencing of internal transcribed spacer (ITS)/D1-D2 Large Subunit (LSU) regions. Chromagar Candida Plus has been developed to improve culture-based detection, appearing as light blue colonies with a blue halo. Rapid and accurate diagnosis is critical for outbreak control [7].

*C. auris* outbreaks have been reported worldwide, particularly in intensive care settings. Genomic epidemiology confirms clonal transmission within hospitals but distinct clades globally. Environmental persistence and skin colonization are central to its epidemiological success. *C. auris* is readily transmitted, so surveillance and genomic tracking remain vital for outbreak investigation.

*C. auris* causes invasive infections such as bloodstream and wound infections. Case fatality rates can reach 30–60% in vulnerable patients. Unlike most *Candida* species, *C. auris* readily transmits in healthcare settings, contaminating surfaces and medical devices as resilient biofilms. It also persists on skin, resulting in outbreaks across intensive care units [8].

Resistance is common to fluconazole, variable to amphotericin B and emerging to echinocandins. Pan-resistant strains, although rare, have been identified. Resistance mechanisms include mutations in *ERG11* (azole resistance), *FKS1* (echinocandin resistance), and increased efflux pump activity [9]. Limited treatment options necessitate careful antifungal stewardship and consideration of novel agents, such as ibrexafungerp and rezafungin.

Eradication is challenging due to survival and persistence on surfaces for weeks and tolerance to many disinfectants through biofilm extracellular matrix production, efflux pumps and enhanced adhesion. Control requires early identification, patient isolation, strict hand hygiene, dedicated patient equipment and environmental cleaning with chlorine- or hydrogen peroxide–based disinfectants [10]. Cohorting of staff and patients is often necessary. National and international health agencies require surveillance and communication.

## Open questions

- What genomic or environmental factors drive the simultaneous emergence of multiple clades?
- How do different clades vary in virulence and resistance profiles?
- What ecological reservoirs and environmental pressures led to its emergence and spread into clinical environments and from these back into the environment?
- Can novel antifungal strategies be developed that improve treatment outcomes?
- How can rapid, low-cost diagnostics be implemented in low-resource settings?

## Abbreviations

ABC: ATP-Binding Cassette
ITS: internal transcribed spacer
LSU: D1-D2 region of large subunit of ribosomal RNA
LSU: Large Subunit
MALDI-TOF: matrix-assisted laser desorption/ionization–time of flight
SNP: single nucleotide polymorphism
WHO: World Health Organization
